# Comparison of Chemical and Mechanical Prophylaxis of Venous Thromboembolism in Nonsurgical Mechanically Ventilated Patients

**DOI:** 10.1155/2015/849142

**Published:** 2015-11-22

**Authors:** Dany Gaspard, Karen Vito, Christa Schorr, Krystal Hunter, David Gerber

**Affiliations:** ^1^Division of Pulmonary and Critical Care Medicine, Cooper University Hospital, Camden, NJ 08103, USA; ^2^Cooper Research Institute, Cooper University Hospital, Camden, NJ 08103, USA; ^3^Division of Critical Care Medicine, Cooper University Hospital, Camden, NJ 08103, USA

## Abstract

*Background.* Thromboembolic events are major causes of morbidity, and prevention is important. We aimed to compare chemical prophylaxis (CP) and mechanical prophylaxis (MP) as methods of prevention in nonsurgical patients on mechanical ventilation.* Methods.* We performed a retrospective study of adult patients admitted to the Cooper University Hospital ICU between 2002 and 2010. Patients on one modality of prophylaxis throughout their stay were included. The CP group comprised 329 patients and the MP group 419 patients. The primary outcome was incidence of thromboembolic events.* Results.* Acuity measured by APACHE II score was comparable between the two groups (*p* = 0.215). Univariate analysis showed 1 DVT/no PEs in the CP group and 12 DVTs/1 PE in the MP group (*p* = 0.005). Overall mortality was 34.3% and 50.6%, respectively. ICU LOS was similar. Hospital LOS was shorter in the MP group. Multivariate analysis showed a significantly higher incidence of events in the MP prophylaxis group (odds ratio 9.9). After excluding patients admitted for bleeding in both groups, repeat analysis showed again increased events in the MP group (odds ratio 2.9) but this result did not reach statistical significance.* Conclusion*. Chemical methods for DVT/PE prophylaxis seem superior to mechanical prophylaxis in nonsurgical patients on mechanical ventilation and should be used when possible.

## 1. Introduction

Venous thromboembolic events (VTE), mainly deep vein thrombosis (DVT) and pulmonary embolism (PE), are significant medical problems and major causes of morbidity and mortality. They are among the most preventable complications in hospitalized patients, resulting in tens of billions of dollars yearly in healthcare costs [1].

Several techniques for prevention of VTE are clinically utilized including mechanical prophylaxis (MP) (Graduated Compression Stockings (GCS) and Intermittent Pneumatic Compression (IPC) devices) and chemical prophylaxis (CP), the most commonly used regimens being subcutaneous unfractionated heparin (UFH) and low molecular weight heparin (LMWH).

Although various methods of VTE prophylaxis have been compared in a number of patient populations, to the best of our knowledge there have been no studies to date directly comparing the efficacy of mechanical and chemical prophylaxis in preventing these events in nonsurgical patients. For this reason, we chose to evaluate the efficacy of these 2 methods in mechanically ventilated, critically ill patients.

## 2. Material and Methods

We performed a retrospective review of data collected on critically ill, nonsurgical patients admitted to the 30-bed multidisciplinary intensive care unit (ICU) at Cooper University Hospital in Camden, New Jersey. Data was obtained from medical records and from the Project Impact database, which contains demographic, acuity, diagnostic, laboratory, pharmacologic, and clinical data on all patients admitted to the ICU. Information was reviewed and analyzed on patients admitted from October 2002 through August 2010. Institutional Review Board approval was obtained; informed consent was waived. The primary endpoint of the study was the development of deep venous thrombosis or pulmonary embolism.

Data collected included the following baseline demographic and clinical data: age, gender, race, and comorbid conditions (active malignancy, history of DVT/PE, hypertension (HTN), chronic obstructive pulmonary disease (COPD), congestive heart failure (CHF), coronary artery disease (CAD), chronic kidney disease (CKD), and history of cerebrovascular accident (CVA)), as well as acuity (APACHE II score).

Outcome data included ICU length of stay (LOS), hospital LOS, overall mortality, number of days on mechanical ventilation, need for packed red blood cell (PRBC) transfusion, and number of units of PRBC transfused.

*Inclusion Criteria.* Inclusion criteria include medical patients 18 years of age or greater who remained mechanically ventilated in the ICU for more than 48 hours and who received VTE prophylaxis with one of the following modalities: subcutaneous low molecular weight heparin (LMWH), subcutaneous unfractionated heparin (UFH), or Intermittent Pneumatic Compression (IPC) devices.

*Exclusion Criteria.* Exclusion criteria include patients younger than 18 years of age, patients not mechanically ventilated or taken off mechanical ventilation within less than 48 hours, patients admitted to the ICU after surgery, patients who were treated with two or more of the above-mentioned therapies during the same ICU admission, patients treated with therapeutic anticoagulation on admission or before reaching the primary endpoint, patients with a known hypercoagulable state, or patients on chronic warfarin therapy.

*Treatment Groups.* Patients were categorized as falling into 2 treatment groups for the purposes of statistical analysis. Group 1 included patients who had received chemical prophylaxis (i.e., either subcutaneous UFH or LMWH, the available form of the latter used at our institution being enoxaparin). Group 2 included patients who received mechanical prophylaxis (IPC at our institution).

*Secondary Evaluation.* Admission diagnoses were obtained for all patients. Admission ICD-9 codes were used to divide patients into 2 categories: bleeding (patients admitted for any bleeding disorders) versus nonbleeding (all other patients). Prevalence of bleeding was compared between the 2 groups. Then patients admitted for bleeding were removed and statistical comparison of nonbleeders in both groups was done. APACHE II scores were compared between patients admitted for bleeding and all other patients.

## 3. Statistical Analysis

Initial results were compared by univariate analysis of the 2 groups. Normally distributed continuous variables were compared by independent *t*-test. The Mann-Whitney *U* test was used to compare the means of continuous variables that were without normal distributions. The Pearson's chi square test was used to compare the proportions of dichotomous or categorical variables.

Subsequently, a logistic regression multivariate analysis of the results was performed with correction for demographics, comorbid conditions, presence of a central venous catheter, and use of PRBC transfusion, with DVT and/or PE as the dependent variable.

The analysis was performed using SPSS 15.0.1 software.

## 4. Results

Review of the database identified 11,686 admissions to the ICU from October 1, 2002, through August 31, 2010. Of these, 7696 were admitted for medical/nonsurgical indications; a total of 1998 of these patients were mechanically ventilated for 48 hours or more.

After analysis, 748 patients met the inclusion criteria. 329 were treated with chemoprophylaxis: 267 received subcutaneous UFH and 62 had received subcutaneous LMWH. The remaining 419 were treated with IPC devices. Of the subjects excluded, 3 were removed because of preexisting hypercoagulable states (2 patients with Factor V Leiden, 1 patient for history of antiphospholipid syndrome), 126 for being on Coumadin, 28 for being on Argatroban, 391 for being on a continuous heparin infusion, 49 for not being on any prophylaxis method, and 653 for being on multiple prophylactic methods during their hospital stay.

A total of 14 patients experienced a VTE during their ICU stay, 1 in the CP group and 13 in the MP group.

### 4.1. Univariate Analysis

Overall the two groups were similar in terms of demographics and acuity (Tables 1 and 2). Patients receiving CP were more likely to have a higher number of comorbidities than those receiving MP, specifically previous history of DVT/PE, CHF, COPD, and diabetes (Table 2).

Patients receiving chemical prophylaxis had significantly fewer VTE than those receiving mechanical prophylaxis. They also had lower mortality and transfusion requirements, but longer hospital LOS (Table 2).

### 4.2. Multivariate Analysis

When the multivariate regression was performed, accounting for age, hypertension, active malignancy, history of DVT/PE, COPD, DM, CVA, presence of central venous catheter, and PRBC transfusion, individuals receiving MP were more likely to develop a thromboembolic event (odds ratio: 9.9, *p* = 0.028) compared to those receiving CP. Primary endpoint and other study endpoints of the 2 groups are shown in Table 3.

### 4.3. Secondary Analysis

There was a significantly higher proportion of patients admitted to the ICU for bleeding in the MP group (2 patients (0.61%) in the CP group VS 150 patients (35.80%) in the MP group; *p* < 0.01).

Multivariate analysis was repeated after these patients were excluded from both groups (with 327 remaining in the CP group and 269 in the MP group). Results favored an increased number of events in the MP group (odds ratio 2.9) but this result did not meet statistical significance (*p* = 0.358).

APACHE II scores of the 152 patients admitted with bleeding were compared to scores of all other patients; there was no significant difference (*p* = 0.27).

## 5. Discussion

Thromboembolic events in hospitalized patients have historically been relatively common but are also a largely preventable morbid condition. The incidence of thromboembolic events is estimated to be around 1 to 2 million cases every year in the United States, with half to two-thirds of these cases being hospital-acquired. Annual healthcare costs associated with the diagnosis and treatment of these conditions, as well as loss of productivity attributable to them, are substantial although widely disparate, estimated at 2.5–19 billion dollars a year, with some estimates going as high as 55 billion dollars [1]. It has been estimated that half or more of these could have been prevented [2], indicating the importance of raising awareness of this problem and the need to institute more rigorous protocols for their prevention.

Thromboembolic events in the ICU are a common occurrence. In 1995, Hirsch et al. found that 33% of ICU patients developed DVT during or after their ICU stay [3]. In a study by Crowther et al. where patients were screened for the presence of clinically silent DVT and PE, the incidence was approximately 15% [4]. Sud et al. demonstrated that increasing adherence to DVT prophylactic methods resulted in decreased events and was more cost-effective than a routine screening program [5].

It is known that surgery increases the propensity for thromboembolic events largely by inducing a proinflammatory state, and mechanical methods have shown a substantial benefit when used by themselves or as an adjunct to chemical prophylaxis in the postsurgical setting [6, 7]. In contrast, while many nonsurgical patients also have proinflammatory physiology during the course of their illnesses, no studies have compared mechanical and chemical prophylactic methods in these patients, as is noted in the 2012 American College of Chest Physicians guidelines for DVT prophylaxis [8]. Conclusions and recommendations regarding prevention of VTE made by the ACCP have come from extrapolation of surgical data. For this reason we elected to study only nonsurgical patients, in an attempt to determine whether one method of prophylaxis was superior to the other in that population.

Some authors have suggested that one of the shortcomings of mechanical prophylaxis including GCS and IPC is a lack of compliance of patients with these methods. Patients have a tendency to remove them because of discomfort. However, since we have only included mechanically ventilated patients in the ICU, who likely would be receiving some form of sedation and/or analgesia, we believe that this is not a major consideration in our project. Furthermore, a meta-analysis looking at the difference in efficacy between GCS and IPC concluded that there is weak evidence of superiority of IPC over GCS [9]. Since our institution primarily uses IPC devices, the end-result of better efficacy with chemical prophylaxis can be extrapolated to GCS as well.

More recent data suggests that there may be some benefit from adjusting DVT prophylaxis methods depending on the patient's risk of embolic events [10]. Estimating an individual's risk of bleeding is a complex undertaking, and generalization of data for a large population is difficult. At our institution during the study period, no specific protocol was instituted for DVT prophylaxis upon admission, and decisions were left to the admitting physician's clinical acumen. Risk factors accounted for in the regression analysis included age, active malignancy, history of DVT/PE, diabetes mellitus, essential hypertension, stroke, and COPD, as well as presence of a central venous catheter. These factors include all conditions that have either a strong or moderate association with development of DVT/PE (except for use of contraceptive and hormonal therapy) [11]. Some conditions with weak association (such as BMI/obesity, pregnancy and varicose veins) were not available, but we do not believe this is a major impediment to the conclusions of this study.

Patients who received both chemical and mechanical prophylaxis represent an interesting population. Unfortunately when we tried to analyze their results, we found that some had received mechanical and chemical prophylaxis simultaneously, while others received them sequentially, with different durations, causing this population to be very heterogeneous and making it impossible to adjust for those variables. Thus we excluded patients who had gotten more than one modality of prophylaxis to avoid the confounding effect on the interpretation of the outcomes studied.

Basic demographics of the two groups were comparable. Medical acuity, as measured by the APACHE II score, was comparable between the two groups. There were some differences in comorbid conditions between the two groups, namely, a higher percentage of patients with history of DVT/PE, DM, CHF, and COPD in the chemical prophylaxis arm. In our final analysis, we corrected for these variables in the regression model.

Analysis of the primary endpoint shows a 0.3% incidence of DVT/PE in the chemical prophylaxis arm and 3.1% incidence in the mechanical prophylaxis arm. After adjusting for all other variables, we found that patients who had received mechanical prophylaxis were less likely to develop thromboembolic events (odds ratio = 9.9).

A major concern of this study related to its retrospective nature is the potential for selection bias, given the much higher incidence of patients admitted for bleeding in the MP group, raising the concern that the 2 groups compared may not be similar. As an attempt to correct for this shortcoming, a secondary analysis was undertaken where patients admitted for bleeding were removed from both groups. Result of the multivariate analysis in this setting again showed an increased risk in the MP group, but results did not meet statistical significance. Since the APACHE II score of patients admitted with bleeding was not different from other patients, we cannot fully explain the change in results when these patients were excluded. While it may just be the decreased number of patients, the difference seems to suggest that patients admitted with bleeding are at increased risk of thrombosis regardless of the overall acuity of their condition.

It is notable that patients in the mechanical prophylaxis group had a significantly higher mortality rate than those in the chemical prophylaxis group, despite comparable overall acuity. While the increased number of VTE events may have contributed to this, a direct causal relationship between these factors cannot be established based on the retrospective nature of the data presented.

We also analyzed PRBC transfusion and how this relates to our results. A greater percentage of patients in the mechanical prophylaxis arm received PRBC transfusions, and the number of units transfused per patient per ICU stay was also significantly higher. This is related to the higher number of patients admitted for bleeding in the MP group. Several studies show increased risks associated with blood transfusions. They increase inflammatory markers, increase propensity to infection, and decrease the host's intrinsic defense mechanisms. It is possible that these factors contributed to the increased mortality seen with the mechanical prophylaxis group. In addition to that, studies have also shown that PRBC transfusions increase the risk of thromboembolic events. In a large recent study from May 2012 done on 22000 patients undergoing resection for colorectal surgery, PRBC transfusion was shown to increase the risk of DVT and PE [12]. In our study, the fact that patients in the mechanical prophylaxis arm have received significantly more transfusions may have contributed to the increased numbers of thromboembolic phenomena.

The main limitation of the study lies in the fact that it is retrospective. This precludes from having a specific protocol to test for presence of thromboembolic disease.

DVTs and PEs in this situation were looked for specifically because of some level of clinical suspicion; as a consequence our findings are most likely relevant to VTE significant enough to induce clinical signs and/or symptoms. It has been shown, however, that these events are underrecognized in the intensive care setting in particular [13]. Nonetheless, there is no evidence that treating nonclinically significant embolic events improves mortality or other outcomes. In addition, as mentioned previously, protocols aimed at improving compliance with DVT prophylaxis methods seem more cost-effective than methods aimed at screening every patient for the presence of these events.

We believe our selected population of mechanically ventilated nonsurgical patients represents a unique group and should be further analyzed and studied. Randomized controlled trials comparing different methods of prophylaxis in this group of individuals are warranted and would help overcome the shortcomings of our study. Baseline evaluation of individual risk of thromboses and bleeding would be possible in such studies, unlike retrospective analyses.

We would also suggest an evaluation of combination prophylaxis, comparing it to single prophylaxis. Surgical data suggests that it may offer some benefit, but again no studies are available on nonsurgical populations.

## 6. Conclusion

Chemical methods of DVT prophylaxis seem to be superior to mechanical methods in mechanically ventilated nonsurgical patients and should be used whenever possible in this population.

## Figures and Tables

**Table 1:** General characteristics of the study population.

	Overall		
	*N*		
Age (mean/SD)	748	58.31	16.51
Gender (*n*/%)	748		
Male		420	56.15%
Female		328	43.85%
Race (*n*/%)	743		
American Indian		3	0.40%
Asian/Pacific Islander		12	1.62%
Black		262	35.26%
Latin/Hispanic		96	12.92%
White		367	49.39%
Other		3	0.40%
APACHE II point sum (mean/SD)	657	20.21	7.48
HTN (*n*/%)	748	447	59.8%
CAD (*n*/%)	748	115	15.4%
CHF (*n*/%)	748	95	12.7%
CVA (*n*/%)	748	92	12.3%
COPD (*n*/%)	748	106	14.2%
DM (*n*/%)	748	224	29.9%

**Table 2:** Baseline characteristics and comparison of the 2 groups.

	Chemical prophylaxis			Mechanical prophylaxis			*p* value
	*N*			*N*			
Age (mean/SD)	329	57.45	16.67	419	58.99	16.37	0.206
Gender (*n*/%)	329			419			
Male		182	55.32%		238	56.80%	
Female		147	44.68%		181	43.20%	0.685
Race (*n*/%)	327			416			
American Indian		2	0.61%		1	0.24%	
Asian/Pacific Islander		4	1.22%		8	1.92%	
Black		132	40.37%		130	31.25%	
Latin/Hispanic		44	13.46%		52	12.50%	
White		144	44.04%		223	53.61%	
Other		1	0.31%		2	0.48%	0.103
APACHE II point sum (mean/SD)	288	19.8	7.78	369	20.53	7.24	0.215
Active cancer	329	33	10%	419	53	12.6%	0.265
History of DVT/PE	329	18	5.5%	419	11	2.6%	0.045
HTN (*n*/%)	329	208	63.2%	419	239	57.0%	0.087
CAD (*n*/%)	329	52	15.8%	419	63	15.0%	0.772
CHF (*n*/%)	329	51	15.5%	419	44	10.5%	0.041
CVA (*n*/%)	329	38	11.6%	419	54	12.9%	0.580
COPD (*n*/%)	329	63	19.1%	419	43	10.3%	0.001
DM (*n*/%)	329	112	34.0%	419	112	26.7%	0.030

**Table 3:** Primary endpoint and other study endpoints of the 2 groups.

	Chemical prophylaxis			Mechanical prophylaxis			*p* value
	*N*	Events		*N*	Events		
DVTonly (*n*/%)	329	1	0.3%	419	12	2.9%	0.008
PE event (*n*/%)	329	0	0.0%	419	1	0.2%	0.375
DVT/PE (*n*/%)	329	1	0.3%	419	13	3.1%	0.005
ICU LOS in days (median/IQR)	329	7.33	4.93–11.86	419	6.85	4.52–11.81	0.380
Hospital LOS in days (median/IQR)	329	15	9.5–24	419	13	8–23	0.007
MV days (Median/IQR)	329	5.2	3.1–9.98	419	5.21	3.03–9.35	0.908
PRBC transfusion (*n*/%)	329	79	24.0%	419	155	37.0%	0.000
PRBC units (median/IQR)	329	2	1–3	155	4	2–6	0.000
Mortality (*n*/%)	329	113	34.3%	419	212	50.6%	0.000

## Abbreviations

CAD: Coronary artery disease
CHF: Congestive heart failure
CKD: Chronic kidney disease
COPD: Chronic obstructive pulmonary disease
CP: Chemical prophylaxis
CVA: Cerebrovascular accident
DVT: Deep vein thrombosis
GCS: Graduated Compression Stockings
HTN: Hypertension
ICU: Intensive care unit
IPC: Intermittent Pneumatic Compression device
LMWH: Low molecular weight heparin
LOS: Length of stay
MP: Mechanical prophylaxis
PE: Pulmonary embolism
PRBC: Packed red blood cell
UFH: Unfractionated heparin
VTE: Venous thromboembolism.
